# Theranostic nanoplatform to target macrophages enables the inhibition of atherosclerosis progression and fluorescence imaging of plaque in ApoE(−/−) mice

**DOI:** 10.1186/s12951-021-00962-w

**Published:** 2021-07-28

**Authors:** Qi Wang, Yong Wang, Siwen Liu, Xuan Sha, Xiaoxi Song, Yue Dai, Mingming Zhao, Lulu Cai, Kai Xu, Jingjing Li

**Affiliations:** 1 School of Medical Imaging, Xuzhou Medical University, Xuzhou, 221006 China; 2 Department of Radiology, Affiliated Hospital of Xuzhou Medical University, Xuzhou, 221004 China

**Keywords:** Atherosclerosis, Theranostic nanomedicines, Macrophage, Fluorescence imaging

## Abstract

**Background:**

Rupture of atherosclerotic plaque can cause acute malignant heart and cerebrovascular events, such as acute coronary heart disease, stroke and so on, which seriously threaten the safety of human life and property. Therefore, the early diagnosis and inhibition of atherosclerotic plaque progress still be a vital task.

**Results:**

In this study, we presented the development of composite mesoporous silica nanoparticle (Ru(bpy)_3_@SiO_2_-mSiO_2_, CMSN)-based nanomedicines (NMs) (Ru(bpy)_3_@SiO_2_-mSiO_2_@SRT1720@AntiCD36, CMSN@SRT@Anti) for accurate diagnosis and treatment of atherosclerosis (AS). In *vitro* cell experiments showed that both RAW264.7 and oxidized low density lipoprotein (ox-LDL)-stimulated RAW264.7 cells could significantly uptake CMSN@SRT@Anti. Conversely, little fluorescence signal could be observed in CMSN@SRT group, showing the excellent targeting ability of CMSN@SRT@Anti to Class II scavenger receptor, CD36 on macrophage. Additionally, such fluorescence signal was significantly stronger in ox-LDL-stimulated RAW264.7 cells, which might benefit from the upregulated expression of CD36 on macrophages after ox-LDL treatment. For another, compared with free SRT1720, CMSN@SRT@Anti had a better and more significant effect on the inhibition of macrophage foaming process, which indicated that drug-carrying mesoporous silicon with targeting ability could enhance the efficacy of SRT1720. Animal experimental results showed that after the abdominal injection of CMSN@SRT@Anti, the aortic lesions of ApoE-/-mice could be observed with obvious and persistent fluorescence signals. After 4 weeks post-treatment, the serum total cholesterol, aortic plaque status and area were significantly improved in the mouse, and the effect was better than that in the free SRT1720 group or the CMSN@SRT group.

**Conclusions:**

The designed CMSN@SRT@Anti with excellent biocompatibility, high-performance and superior atherosclerosis-targeting ability has great potential for accurate identification and targeted therapy of atherosclerotic diseases.

**Graphic abstract:**

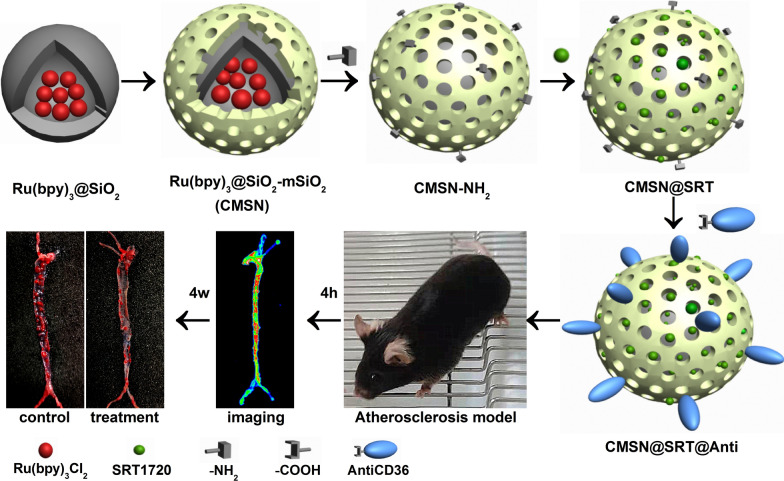

**Supplementary Information:**

The online version contains supplementary material available at 10.1186/s12951-021-00962-w.

## Background

Cardiovascular disease (CVD) is a widely recognized human killer, and as a representative CVD, coronary heart disease (CHD) has a very high mortality rate, accounting for more than half of the deaths of CVD cases worldwide every year [1, 2]. AS is the basic lesion of CVD. With the progression of AS, the rupture of gradually enlarged plaque can lead to vascular stenosis or occlusion, causing CHD and stroke [3]. Therefore, the accurate identification and effective intervention of AS is of great significance in preventing the occurrence of life-threatening events and disabilities.

NMs can make use of the characteristics of disease composition to cleverly identify and interfere with the outcome of disease. The well designed NMs have been widely applied in various diseases such as the diagnosis and therapy of tumor, cardiovascular disease [4], the inhibition of bacterial infection [5, 6], bone regeneration [7] and so on. For example, mitochondria are essential for cancer cells to make DNA. Therefore, mitochondrial-targeting NMs loaded with immunomodulator (R848) were designed to effectively inhibit cancer recurrence and metastasis through combination photothermal therapy and immune checkpoints blockade [8]. Photosensition-free NMs filled with nitric oxide (NO) donors were reported to promote mitochondrial damage and DNA fragmentation in cancer cells, thereby initiating programmed cancer cell death [9]. Similarly, AS has its own characteristics, which have also been utilized for the diagnosis and therapy of AS. The pathological process of AS is complex and involves many pathological mechanisms. In essence, it is a process of progressive aggravation of chronic inflammation, which is characterized by the deposition of lipids and various inflammatory cells under arterial intima [10, 11]. Ox-LDL is an important participant in the occurrence and development of AS. Macrophages phagocyte ox-LDL through scavenger receptors and then transform into foam cells [12]. Based on this mechanism, some diagnosis and therapy complex have been fabricated for AS. IK17 (the first human single chain FV antibody fragment) was reported to be able to bind with the lipid and protein regions of ox-LDL and inhibit the phagocytosis of ox-LDL by macrophages. Shaw Px et al. injected iodinated IK17 (125I-IK17) into AS mice and realized the precise therapy for AS with ox-LDL as the target [13]. Except ox-LDL, a series targets which play a key role in the development of AS have also been chosen for the specific diagnosis and therapy of AS. Macrophages can mediate the circulation of inflammatory cells in AS lesions and promote the formation of AS plaques by summoning fibroblasts. Thus, macrophage as the target was employed to diagnosis and treatment of AS. The transporter protein (TSPO) is significantly expressed on the surface of macrophages, and TSPO ligand iodine 125-DPA-713 prepared by Foss CA et al. can efficiently accumulate in AS lesions, thus achieving the molecular diagnosis and treatment of AS with macrophages as the target [14]. Because the proliferation and migration of vascular smooth muscle cells (VSMCs) present in AS lesions is related to the formation of fibrous cap, profilin-1 highly expressed on the surface of VSMCs was also chosen as the target [15]. Zhang SH et al. covalently combined Profilin-1 Antibody (PFN1) with paramagnetic Fe_3_O_4_ to obtain near-infrared fluorescence imaging/MRI nanoprobe, which could locate AS plaques by imaging and moderate the progression of AS [16]. In the early stage of AS, neutrophils appear in the lesions of AS, which was used as molecular targets for the ultra-early diagnosis of AS. Elastic cell enzyme exists in neutrophils plasmids and can be employed as target to indicate the presence of neutrophils. Glinzer A et al. prepared a near-infrared imaging agent Neutrophil Elastase 680 FAST to target elastic cell enzyme to realize the early diagnosis of AS [17]. In the late stage of AS, fibrin deposits on the plaque surface. Therefore, fibrin as a molecular target was introduced for the diagnosis of advanced AS. Replacing the DE loop of Simian virus 40 (SV40) major capsid protein VP1 with CREKA peptide which can target fibrin, Sun X et al. fabricated trifunctional SV40-based nanoparticles with QD800 encapsulation. Near-infrared fluorescence imaging shows that AS plaques at the late stage in ApoE (−/−) mice could be noninvasively fluorescently imaged [18].

**Scheme 1:**
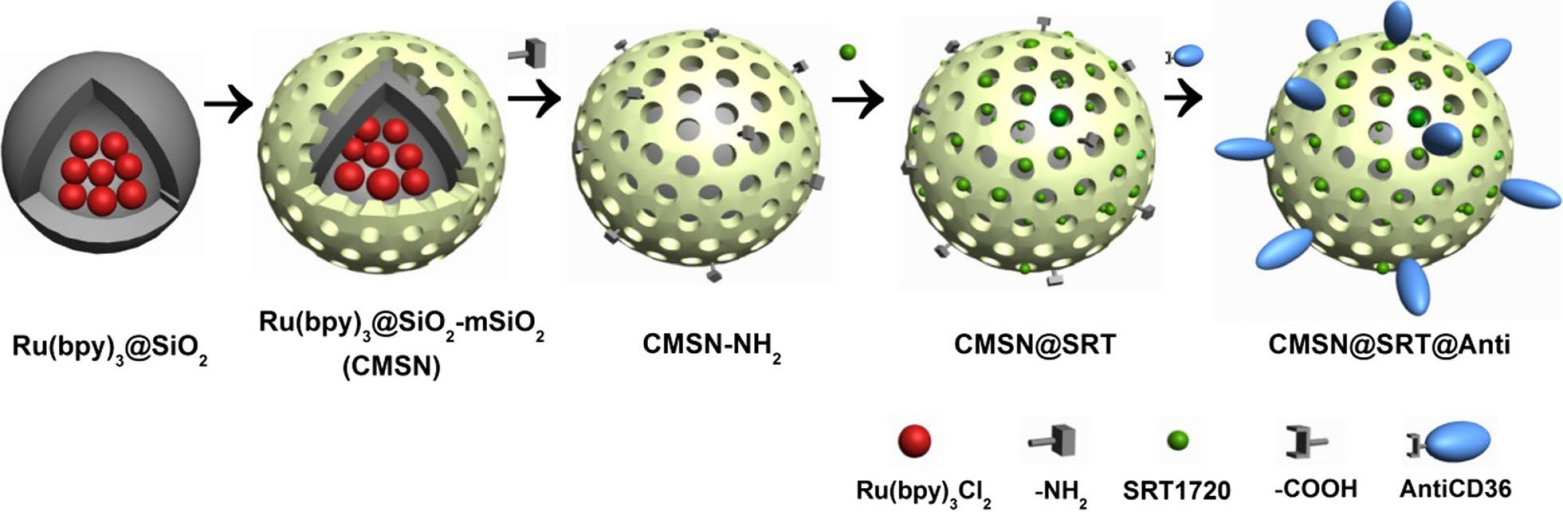
Schematic illustration of CMSN@SRT@Anti formation process. The fluorescent CMSN was used as nanocarrier to load SRT1720 and conjugate with AntiCD36 to obtain the theranostic nanomaterials

The above successful examples for the molecular diagnosis and treatment of AS stimulates us to explore new probe to improve the theranostic functions. In this study, we selected CD36 as a target for the molecular diagnosis and treatment of AS. Studies have shown that CD36, which is expressed on the surface of macrophage, is a key regulatory point for the formation and development of AS. It can mediate the conversion of macrophage into foam cell after phagocytosis of ox-LDL [19, 20]. AntiCD36 can specifically bind to CD36 and inhibit macrophage phagocytosis of ox-LDL [21]. In order to strengthen the treatment effect on AS, SRT1720 was also introduced. As a specific activator of SIRT1, SRT1720 is 1000 times more active than resveratrol [22]. SRT1720 can mediate vasodilation, down-regulate the expression of pro-inflammatory cytokines, promote the reverse transport of cholesterol in macrophages, inhibit macrophage foaminess and the expression of matrix metalloproteinases (MMPs), as well as prevent thrombosis and stabilize plaques [23–25]. To load SRT1720, CMSN were employed as the nanocarrier and conjugated with AntiCD36 to target macrophages in AS plaque. The synthetic process of CMSN@SRT@Anti was illustrated in Scheme 1. With the fluorescence imaging of Ru(bpy)_3_Cl_2_ and the targeted binding ability of AntiCD36 to CD36 overexpressed on the surface of macrophage, we could track the AS plaque and the inhibition effect of AS progress specifically both in *vitro* and in *vivo*.

## Results

### Structure and characteristics of NMs

**Fig. 1:**
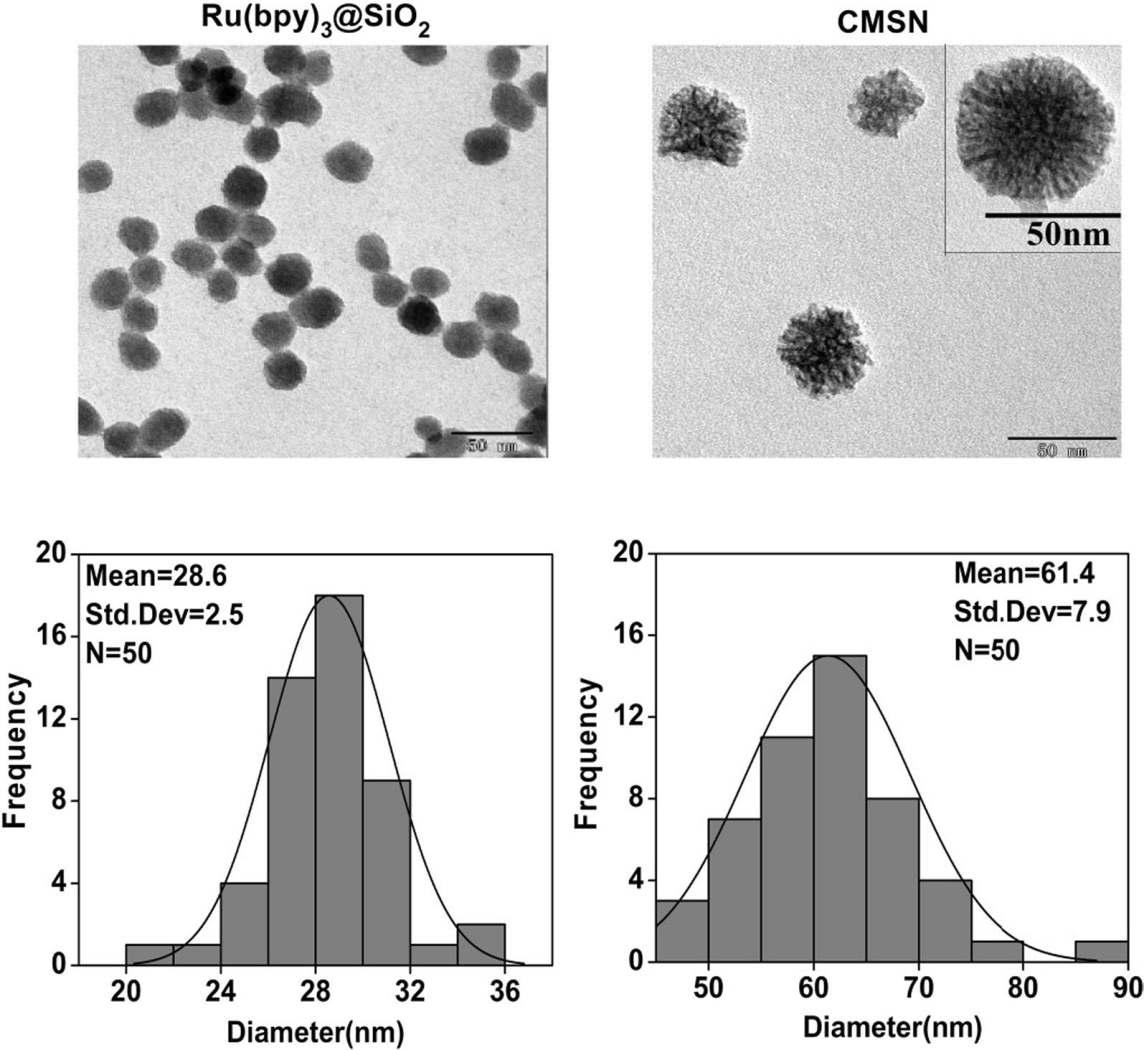
TEM images (Scale bar: 50 nm) and size distributions of Ru(bpy)_3_@SiO_2_ and CMSN

**Fig. 2:**
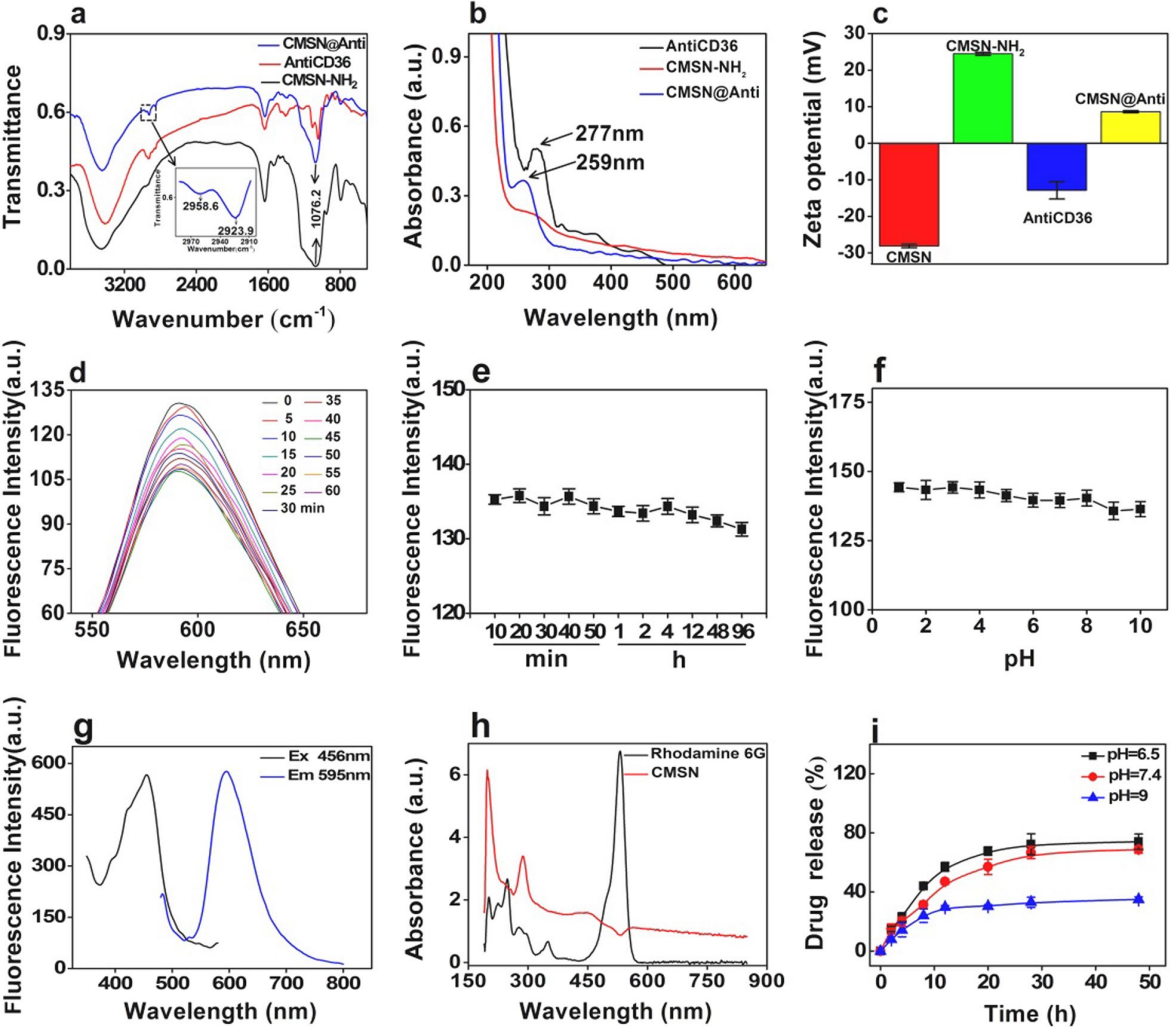
Structure and characteristics of NMs. **a-c** FT-IR, UV–vis and zeta potential characterization to show the formation of CMSN@Anti. **d-f** The fluorescence stability of CMSN under 365 nm UV exposure, natural light and different pH buffers. **g** The fluorescence excitation and emission spectra of CMSN. **h** The UV–vis absorption spectra of CMSN and Rhodamine 6G. **i** The drug release curve of CMSN@SRT@Anti in PBS buffers with different pH values

The morphology of prepared Ru(bpy)_3_@SiO_2_ and CMSN were observed by Transmission Electron Microscopy (TEM). As shown in Fig. 1, spherical structure for Ru(bpy)_3_@SiO_2_ with an average size of 28.6 ± 2.5 nm and mesoporous structure for CMSN with an average size of 61.4 ± 7.9 nm were displayed with well dispersion. Fourier transform infrared spectra (FT-IR), ultraviolet–visible (UV–vis) absorption spectra and zeta potential determination were employed to monitor the whole assembly procedure of CMSN@Anti (Fig. 2a–c). FT-IR showed that CMSN@Anti had the -CH of AntiCD36 at the peak of 2958.6 cm^−1^ and 2923.9 cm^−1^. UV–vis absorption spectra displayed that the characteristic absorption peak of AntiCD36 at 259 nm was observed in CMSN@Anti. Compared with the absorption peak of AntiCD36 at 277 nm, a blue shift of absorption peak of AntiCD36 in CMSN@Anti might be caused by the change of the force between adjacent particles after the binding of nanoparticles to Antibody [26]*.* The zeta potential of CMSN was − 28.1 ± 0.5 mV and was changed to 24.5 ± 0.4 mV after amidation, as well as 8.6 ± 0.3 mV after further AntiCD36 conjugation because of the negative charged AntiCD36 (− 12.8 ± 2.4 mV). All of these characterizations indicated that AntiCD36 had been successfully attached to the surface of CMSN.

To be further used as a fluorescent agent, their fluorescence property was evaluated (Fig. 2d–f). The results of photobleaching resistance of CMSN under 365 nm UV lamp for different time showed that only a 15% fluorescence loss of CMSN after 60 min 365 nm UV exposure. At room temperature, CMSN still maintained a strong fluorescence signal after 96 h under natural light. In addition, the fluorescence signal of CMSN was relatively stable at different pH values from 1 to 10, and no obvious fluorescence quenching phenomenon was observed. It should be mentioned that compared with Ru(byp)_3_Cl_2_, CMSN presented better fluorescence stability under 365 nm UV exposure (Additional file 1: Fig. S1a). On the other hand, the hydrodynamic diameter of CMSN@Anti changed litter after storage of 9 days at room temperature, showing their excellent stability and potentials for biomedical applications. The average hydrodynamic diameters were determined to be 225.3 nm, 231.6 nm, 267.8 nm and 255.4 nm at the first, second, fifth and ninth day (Additional file 1: Figure S1b). The excitation and emission spectrum of CMSN was shown in Fig. 2g and its fluorescence quantum yield was determined to be 0.52 by Williams gradient method [27] with Rhodamine 6G as the reference (Fig. 2h).

Additional file 1: Figure S2a, b shows the UV–vis absorption spectra of SRT172s0 at different concentrations and their corresponding linear fitting graphs. According to the calculation, the encapsulation efficiency (EE) and loading efficiency (LE) of CMSN@SRT@Anti were determined to be 47 ± 4% and 42 ± 2% when the concentration of SRT1720 was 3 mM (Additional file 1: Table S1). The drug release curve of CMSN@SRT@Anti in different pH values of PBS was shown in Fig. 2i. The effect of drug release in neutral and weak acidic environment is stronger than that in alkaline environment. Under neutral or weak acid conditions, the SRT1720 release could reach 69 ± 3% or 74 ± 5% for 48 h, respectively.

### Cytotoxicity assessment of CMSN@SRT@Anti

Macrophages, as the core cells of AS, are important components involved in the occurrence and development of AS. In this study, mouse macrophage cell line, RAW264.7 was used as the experimental object. Prior to study the targeted fluorescence imaging and macrophage foaming inhibition of CMSN@SRT@Anti, thiazole blue tetrazole bromination (MTT) assay was first introduced to evaluate their cytotoxicity. As shown in Additional file 1: Figure S3, the survival rate of RAW264.7 or NIH-3T3 cells were not significantly reduced even when the concentration of CMSN@SRT@Anti was 40 μg/mL. One-way ANOVA was used to analyze the absorbance values of each treatment group (*F*_RAW264.7_ = 1.679, *P* = 0.142 > 0.05; *F*_NIH-3T3_ = 1.737, *P* = 0.128 > 0.05). Therefore, a concentration of CMSN@SRT@Anti below 40 μg/mL was chosen for the following cell imaging and the evaluation of inhibition effect of macrophage foaming.

### In vitro targeting ability and fluorescence imaging of CMSN@SRT@Anti on macrophages

**Fig. 3:**
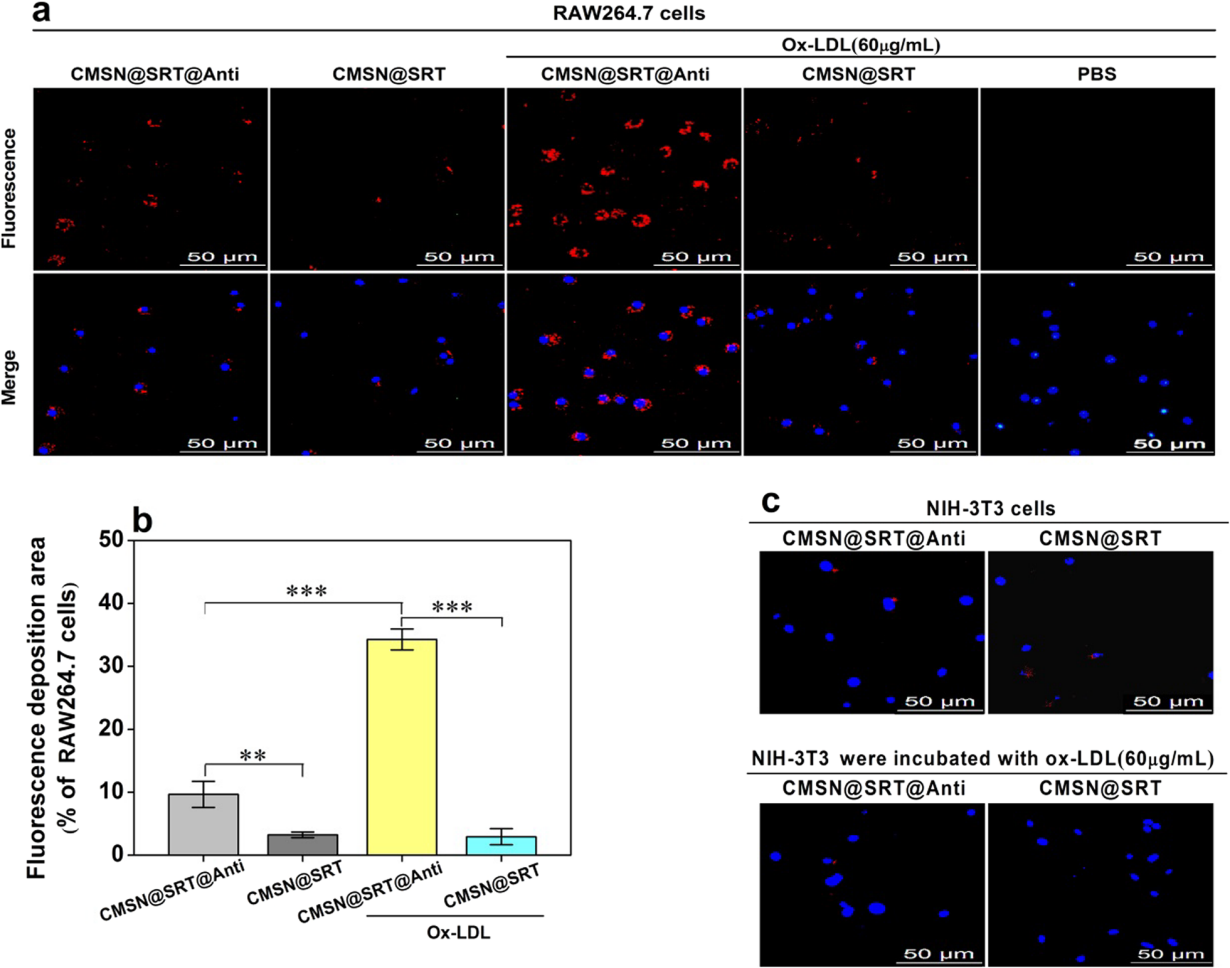
Targeting property of NMs to cells. **a** Representative pictures of NMs targeting RAW264.7 cells. **b** Quantification of fluorescence area in RAW264.7 cells (**P < 0.01, ***P < 0.001). **c** Targeting property of NMs on NIH-3T3 cells. The red fluorescence emissions came from Ru(bpy)_3_Cl_2_ in NMs and the blue fluorescence emissions came from the DAPI staining of cellular nucleus. Scale bars, 50 µm

The targeting ability of CMSN@SRT@Anti to macrophage was performed by fluorescence imaging. Both RAW264.7 and ox-LDL-stimulated RAW264.7 cells displayed a strong red fluorescent signal in cytoplasm after interacting with CMSN@SRT@Anti. Conversely, little fluorescence signal could be observed in CMSN@SRT group (Fig. 3a, b), showing the excellent targeting ability of CMSN@SRT@Anti to CD36 on macrophage. Additionally, such cytoplasmic fluorescence signal was significantly stronger for ox-LDL-stimulated RAW264.7 cells (Fig. 3b: ***P < 0.001 vs. unstimulated cells), which might benefit from the upregulated expression of CD36 on macrophages after ox-LDL treatment [20]. The western blot result further showed that the expression of CD36 was increased in ox-LDL-treated RAW264.7 cells (*P* < 0.05 ox-LDL stimulation group vs. control group) (Additional file 1: Fig. S4). Figure 3c showed that there was almost no fluorescent signal could be observed in NIH-3T3 cells. On the whole, our fabricated CMSN@SRT@Anti could target macrophages, especially activated macrophages stimulated by ox-LDL.

### Inhibition on macrophage foaming by CMSN@SRT@Anti

**Fig. 4:**
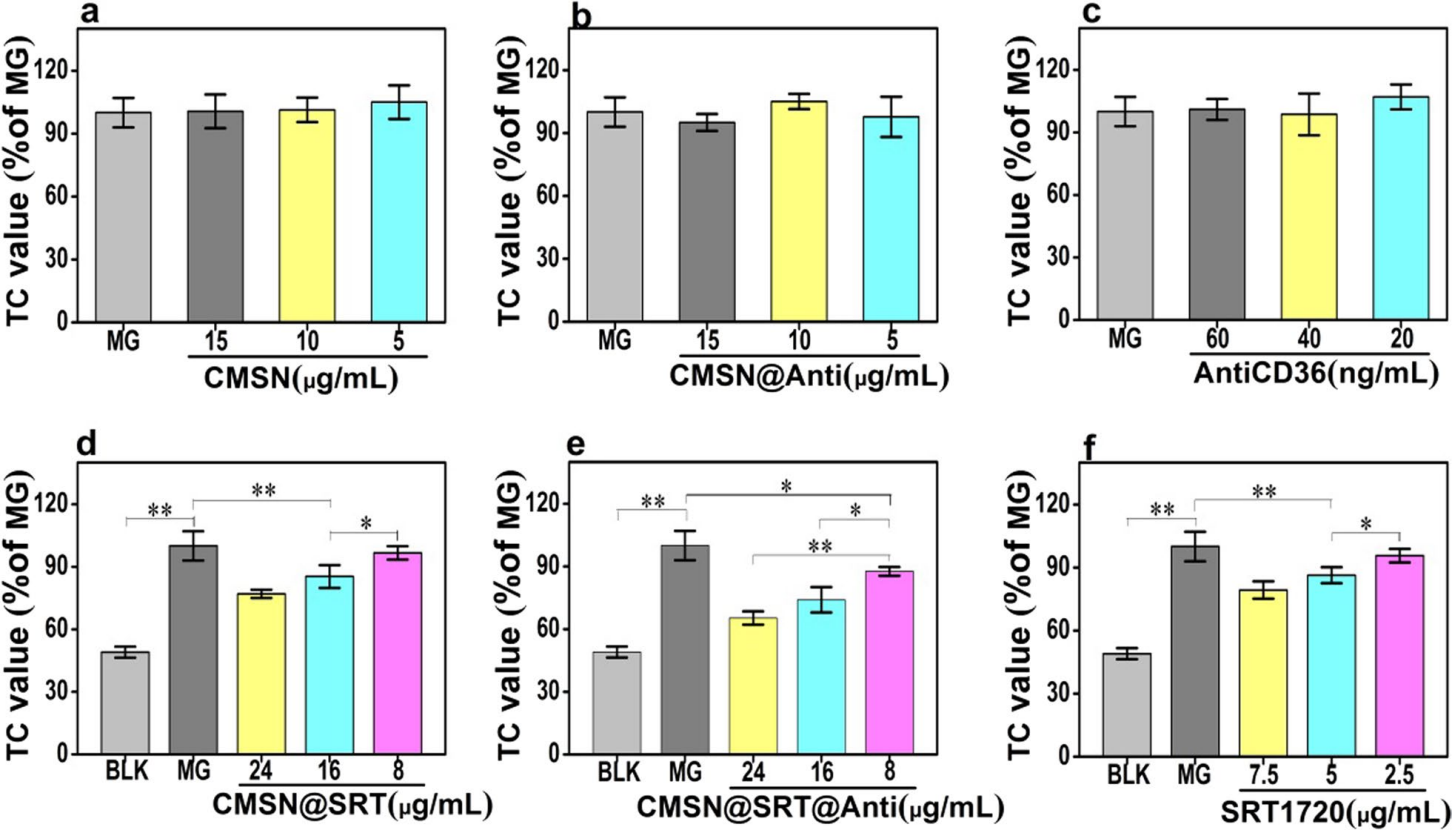
Inhibition effect of different nanomaterials on the foaming process of RAW264.7 cells. **a-f** The effects of CMSN, CMSN@Anti, AntiCD36, CMSN@SRT, CMSN@SRT@Anti or SRT1720 on TC value of RAW264.7 cells (*P < 0.05, **P < 0.01)

**Fig. 5:**
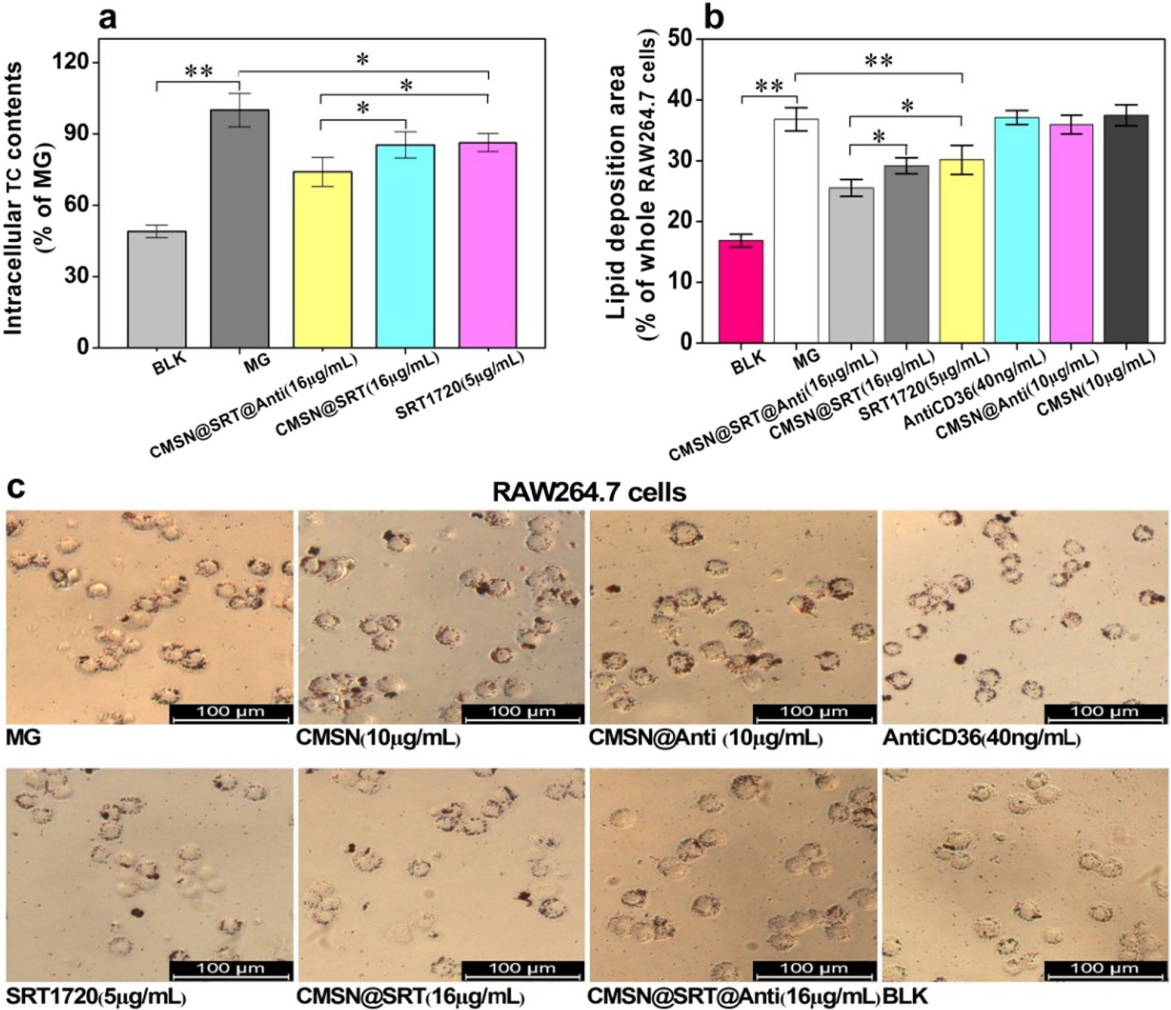
Comparison of RAW264.7cell foaming degree after different intervention. **a** The effects of different treatments on TC value of RAW264.7 cells (*P < 0.05, **P < 0.01). **b** The proportion of Oil Red O staining area in RAW264.7 cells after different treatments (*P < 0.05, **P < 0.01). **c** Representative pictures of Oil Red O staining of intracellular lipid droplets (Scale bar: 100 μm). Note: 16 μg of CMSN@SRT@Anti contained about 10 μg of CMSN, 40 ng AntiCD36, and released about 5 μg SRT1720 within 48 h

The level of total cholesterol (TC) in cells could reflect the degree of lipid deposition to indicate the degree of cell foaming. Compared with the model group (MG), either CMSN, CMSN@Anti or AntiCD36 could reduce the cell TC value little (Fig. 4a–c: *P* > 0.05). Compared with MG, CMSN@SRT at 16 μg/mL or 24 μg/mL could decrease the cell TC value (**P < 0.01), but there was no significant difference in the effect between the two groups (*P* > 0.05) (Fig. 4d). But CMSN@SRT@Anti at 8 μg/mL, 16 μg/mL, or 24 μg/mL could all reduce the cell TC value compared with MG and the effect was enhanced with the concentration increased (Fig. 4e). Free SRT1720 at 5 μg/mL or 7.5 μg/mL could decrease the cell TC value compared with MG (**P < 0.01), but there was no significant difference in the effect between the two groups (*P* > 0.05) (Fig. 4f). With the same SRT1720 concentration, CMSN@SRT@Anti displayed lower intracellular TC contents than free SRT1720 or CMSN@SRT, showing the best inhibition effect of macrophage forming was achieved with the help of AntiCD36 (Fig. 5a).

In order to clearly visually demonstrate these inhibitory effects of each group on macrophage foaming, the treated cells were measured by Oil Red O staining. Similar with the results of TC analysis, the Oil Red O-positive areas in RAW264.7 cells were enhanced after pre-treated with ox-LDL. But they were reduced after co-incubation with free SRT1720, CMSN@SRT or CMSN@SRT@Anti and reached the lowest point for CMSN@SRT@Anti treatment. CMSN, CMSN@Anti or AntiCD36 alone displayed no influence on the macrophage foaming (Fig. 5b, c).

### In vivo toxicity evaluation and metabolism of CMSN@SRT@Anti

**Fig. 6:**
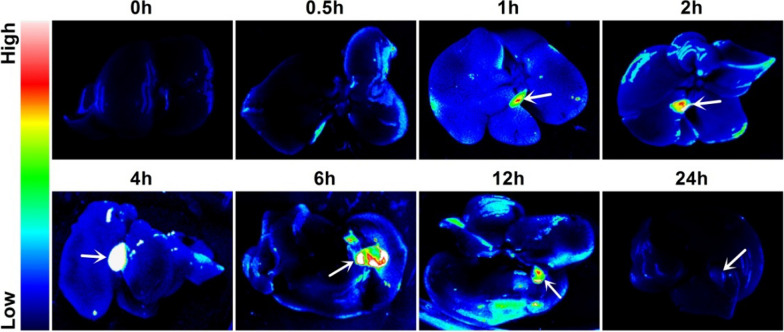
Fluorescence images of liver and cholecyst of Kunming mice at each time point after intraperitoneal injection of CMSN@SRT@Anti (white arrow pointing to cholecyst)

After intraperitoneal injection of CMSN@SRT@Anti to Kunming mice for 1 d, 7 d or 21 d, the blood test results (Additional File 1: Figure S5) showed that compared with the control group, there was no significant difference in the chosen blood indicators (Alanine aminotransferase (ALT), Total protein (TP), albumin (ALB), Creatinine (CREA), urea(UREA), White blood cells (WBC), Red blood cell (RBC), Hemoglobin (HGB), Hematocrit (HCT), Mean Corpuscular Hemoglobin (MCH), Mean red blood cell volume (MCV)) (*P* > 0.05), except a transient increase in Aspartate aminotransferase (AST), which returned to normal level after 7 d. The body weight of the mice showed a slight weight loss on the first day after CMSN@SRT@Anti injection and a gradual increase thereafter, with a growth curve similar to that of the control group (Additional file 1: Figure S6a). Histological examinations of major organs of Kunming mice were performed with intraperitoneal administration of CMSN@SRT@Anti for 1 d and 21d. It showed that no organic lesions, inflammation or other abnormalities were observed in the liver, spleen, heart, kidney and lung of mice (Additional file 1: Figure S6b). To better understand the metabolism of the injected nanomaterials in mice, visceral fluorescence was detected at different time points post-injection. As shown in Fig. 6 and Additional file 1: Table S2, the fluorescence emissions changes from weak-strong–weak in liver and cholecyst with the time passing, indicating the possible excreted route of CMSN@SRT@Anti was through the hepatobiliary systems.

### Specific targeting and progress inhibition of AS plaque in mice

**Fig. 7:**
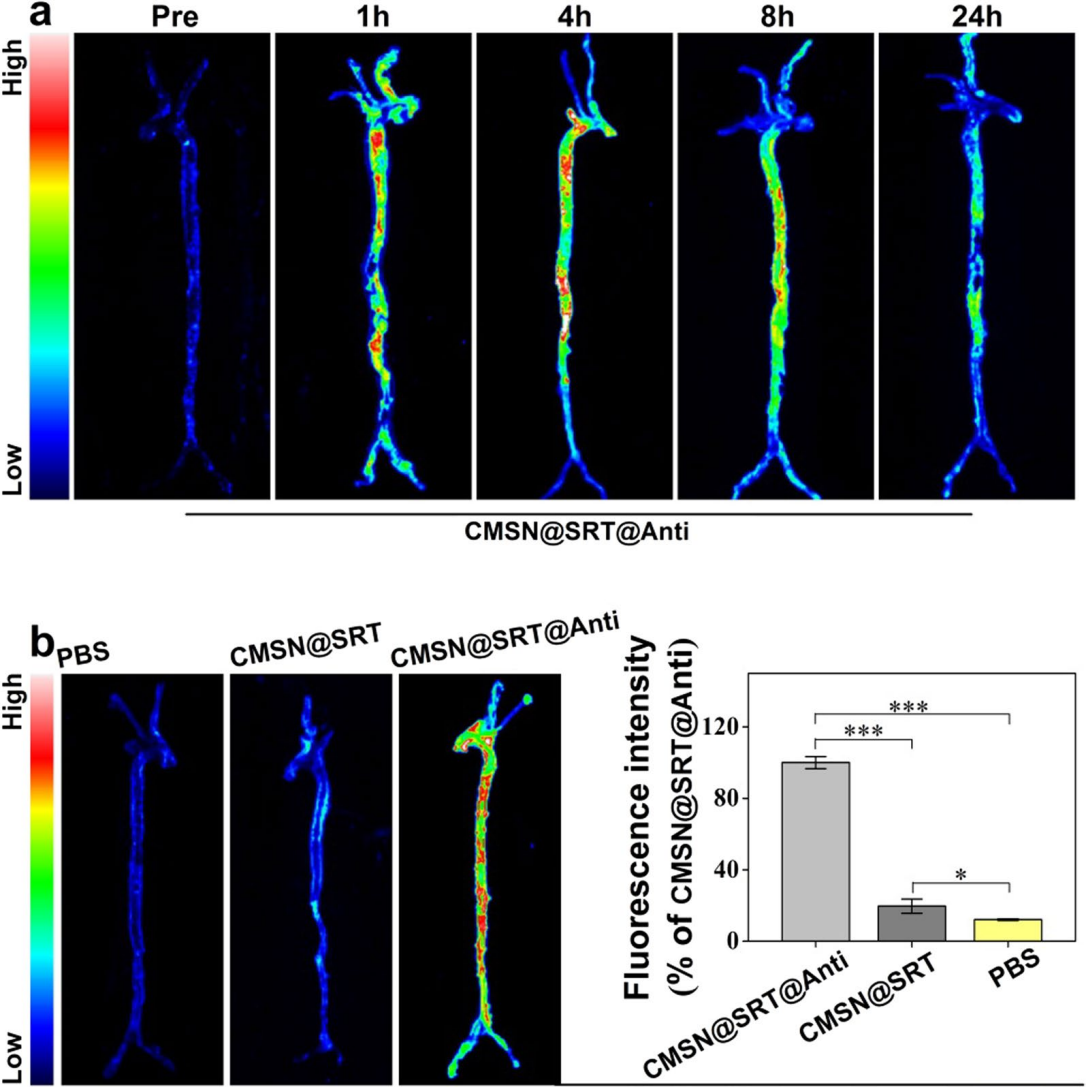
Fluorescence imaging of AS lesions in ApoE−/− mouse aorta. **a** Fluorescence intensity in AS lesion area of mouse aorta at different time points after intraperitoneal injection of CMSN@SRT@Anti. **b** Fluorescence imaging of mouse aorta under different treatments and the corresponding histogram of fluorescence intensity (*P < 0.05, ***P < 0.001)

In order to select the best imaging time point of nanoparticles in vivo, CMSN@SRT@Anti were intraperitoneally injected into AS model mice. The fluorescence imagings of aorta at different time points were collected and shown in Fig. 7a. The results displayed that continuous fluorescence signal was observed in the atherosclerotic lesions of mice after CMSN@SRT@Anti injection, and the strongest signal appeared at time points 1 h, 4 h and 8 h, especially 4 h. Therefore, 4 h was selected as the time point to compare the plaque targeting ability of CMSN@SRT@Anti, CMSN@SRT and PBS. As shown in Fig. 7b, the fluorescence signal in the aorta of mice treated with CMSN@SRT@Anti was significantly higher than that of CMSN@SRT or PBS (***P < 0.001 vs. CMSN@SRT or PBS).

**Fig. 8:**
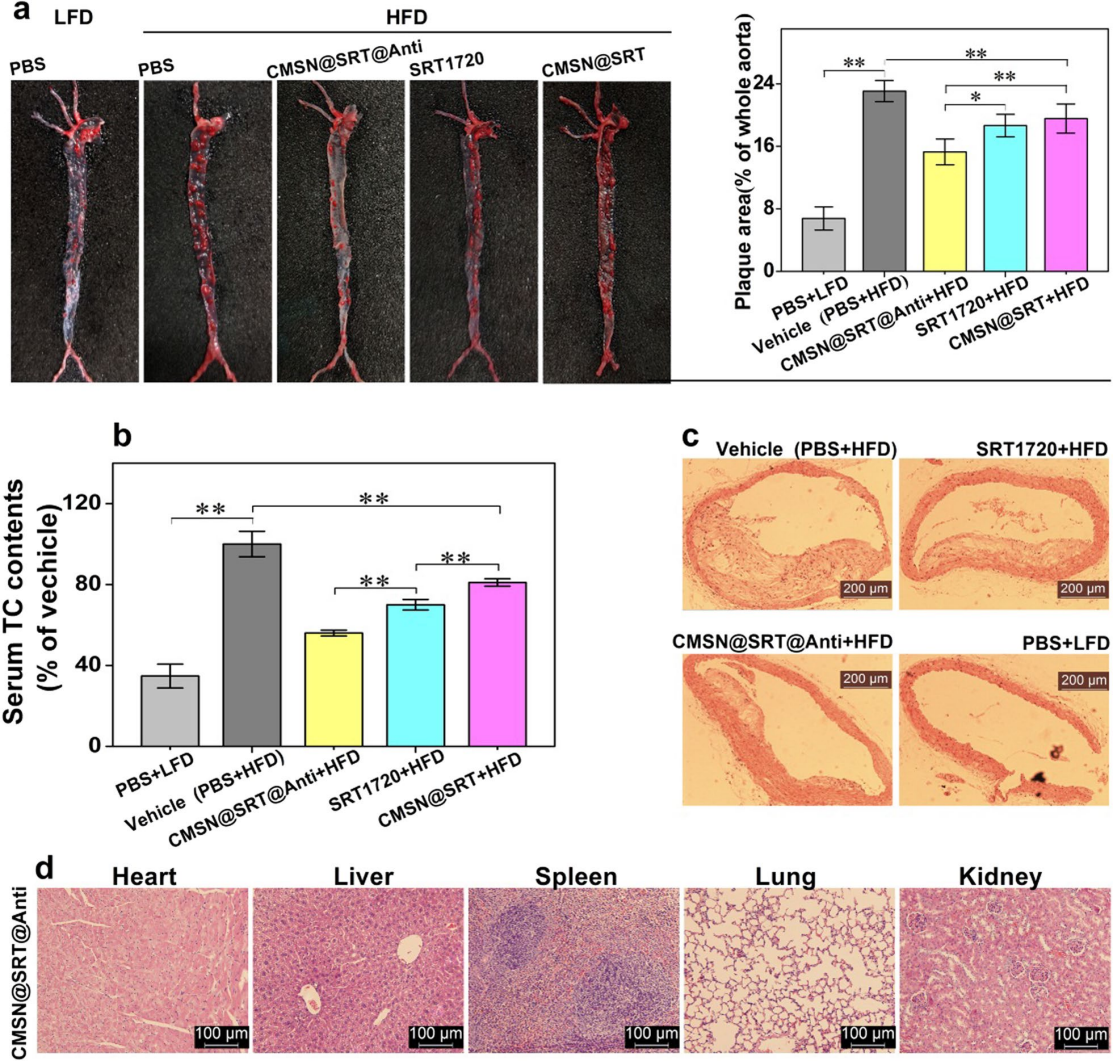
Inhibition effect of different treatments on the progression of AS in ApoE-/-mice. **a** Representative pictures of Oil red O staining of mouse aortic plaque and quantification of Oil Red O-stained area (*P < 0.05, **P < 0.01). **b** Quantitative analysis of TC levels in the mouse serum (**P < 0.01). **c** Pathological section of aorta in mouse (H&E, Scale bar: 200 μm). **d** Pathological section of major organs in mouse (H&E, Scale bar: 100 μm). Note: about 50 mg SRT1720 can be released in 48 h by 170 mg CMSN@SRT@anti or CMSN@SRT

To further visually display the anti-atherosclerosis effect of CMSN@SRT@Anti, the aortas of mice with the intervention were dissected and stained with Oil Red O and the ORO-positive area were quantitatively analyzed (Fig. 8a). This image showed that with the same SRT1720 concentration, CMSN@SRT@Anti, CMSN@SRT or free SRT1720 could all reduce the aorta red staining area of ApoE−/− mice compared with Vehicle group (**P < 0.01). Notably, CMSN@SRT@Anti reduced the area of aorta red stain more effectively than CMSN@SRT (**P < 0.01) or SRT1720 (*P < 0.05), indicating CMSN@SRT@Anti had the best anti-AS effect in vivo. We also carried out quantitative analysis on serum TC values of mice in each group after different treatments. Learnt from Fig. 8b, the injection of CMSN@SRT@Anti, CMSNs@SRT or free SRT1720 can all reduce the blood TC value of AS model mice (**P < 0.01 vs. Vehicle group). Similarly, CMSN@SRT@Anti treatment resulted in a significant decrease in serum TC content than CMSN@SRT or SRT1720 (**P < 0.01).

Histological cross-sections were stained with H&E to further confirm such inhibition effect (Fig. 8c). Compared with the Vehicle group, the proportion of AS plaque area and lumen cross section in mice treated with CMSN@SRT@Anti was significantly reduced. In addition, H&E staining on the main viscera of mice treated with CMSN@SRT@Anti showed that no significant organic lesions, inflammation or other abnormalities were observed in the major organs of ApoE−/−mice after 4 weeks post-injection of CMSN@SRT@Anti, displaying their excellent biocompatibility (Fig. 8d).

## Discussion

At present, NMs have been widely used in the scientific research of various diseases, such as cancer, metabolism-related diseases, etc. [28]. However, NMs for AS is rare. To construct a nanoprobe to target AS plaque and deliver drug for the inhibition of AS progress, Ru(bpy)_3_Cl_2_ doped mesoporous silicon was chosen as the nanocarrier in this work. Mesoporous silicon (MSN) has been widely used in molecular diagnosis and treatment of various diseases and has been recognized as a General Recognized as Safe (GRAS) by the U.S. Food and Drug Administration (FDA) for its non-toxic, stable and biocompatible properties. Hollow MSN is an ideal drug-loading nanoplatform with low density, large specific surface area, strong surface plasticity and large drug loading capacity [29–31]. On the other hand, Ru(bpy)_3_Cl_2_ is widely used in fluorescence labeling due to its good photobleaching resistance and wide detection range. Specifically, when Ru(bpy)_3_Cl_2_ was sealed in silicon spheres, its fluorescence stability can be significantly improved [32]. Thus, based on the selective etching method [30], CMSN as a platform was fabricated to load drug and conjugate with targeting molecule for the diagnosis and treatment of AS. After amination, the solubility of CMSN-NH_2_ was further improved, and the surface charge was changed from − 28.1 ± 0.5 mV to 24.5 ± 0.4 mV, which was consistent with the characterization of mSiO_2_ amination by Dai Y. et al. [33], indicating that CMSN-NH_2_ was successfully prepared. Using Rhodamine 6G as the reference material, the fluorescence quantum yield of CMSN was as high as 52%, indicating that CMSN had a good application prospect as a fluorescent agent. Furthermore, their fluorescence signal still remained at a high level even under the irradiation of 365 nm UV lamp for 1 h, and a stable fluorescence signal under a wide range of pH (1–10) environments, indicating their good anti-photobleaching ability and stability to pH changes, which was consistent with the description of Ru(bpy)_3_@SiO_2_ fluorescence stability by Ho Ta et al. [32]. In this work, Ru(bpy)_3_Cl_2_ was sealed in the silicon sphere, which could effectively block Ru(bpy)_3_Cl_2_ agglomeration. No significant change in hydrated particle size of CMSN@Anti for 9 days standing at room temperature further showed their excellent colloid stability, which was necessary for the biomedical applications.

In recent years, a large number of studies have shown that macrophages play a decisive role in the pathological process of AS. Early ideas suggested that unstable plaques were characterized by a large necrotic center with a large infiltration of macrophages [34, 35]. However, the latest research results indicate that whether macrophages can promote the formation of vulnerable atherosclerotic plaques (VASPs) mainly depends on the polarization state of macrophages rather than the number of macrophages. Under different cytokine environment, macrophages can differentiate into opposite subtypes, namely, pro-inflammatory macrophages (M1) and anti-inflammatory macrophages (M2). M1 macrophages can promote microcalcification deposition in the necrotic center of AS plaques through vesicle-mediated mineralization. M2 macrophages repair and stabilize plaques by inducing vascular smooth muscle cells (VSMCs) to mature [36, 37]. Therefore, the localization of M1-type macrophages has become the key point in the diagnosis and treatment of AS. CD36, expressed on the surface of macrophages, can mediate the uptake of ox-LDL by macrophages, and is a key factor in the polarization of macrophages to M1. It was reported that CD36 inhibitor SSO could significantly inhibit the polarization of macrophages to M1 and make them transform into M2 macrophages [38, 39]. In a recent study by Iryna Voloshyna et al. CD36 was also found to be highly expressed on the surface of M1 macrophages, and the immunosuppressant cyclosporine (CsA) could significantly down-regulate the expression of CD36 on the surface of M1 macrophages [40]. Therefore, in this work, AntiCD36 was modified on the surface of CMSN-NH_2_ to target CD36 for the track and treatment of AS plaque. The fabricated CMSN@Anti was analyzed by Zeta potential apparatus, UV–vis and FT-IR. The existence of specific targeting molecule, AntiCD36 not only favors the track of AS plaque, but also help to improve the drug delivery efficiency and therapy effect eventually. Nicotinamide adenine dinucleotide (NAD +) dependent class III histone deacetylase (SIRT1) has been dubbed as the “longevity gene” [41], which can effectively resist aging, various tumors, cardiovascular diseases, and metabolic diseases. The increased expression of SIRT1 can effectively treat AS. SRT1720, as a SIRT1-specific activator, has been shown to be 1000 times more active than resveratrol [22]. In addition, due to its small molecular weight, SRT1720 is easy to be loaded into nanoparticles, which enables its application to be extended to the field of molecular medicine and further strengthens its therapeutic advantage for AS [42]. In this study, SRT1720 was loaded into the mesopore of CMSN for the treatment of AS. With a 3 mM SRT1720, CMSN@SRT@Anti presented higher LE (42 ± 2%) and EE (47 ± 4%). The release effect of CMSN@SRT@Anti in neutral or weak acidic environment was obviously stronger than that in alkaline, which might come from the good solubility of SRT1720 under weak acid conditions. Similar with chronic inflammation in nature, AS lesions have acidic microenvironment [43]. Nanomaterials with high drug release efficiency in acidic environment have greater therapeutic potential. We also demonstrated the anti-AS superiority of CMSN@SRT@Anti in *vitro* and in *vivo* experiments. In *vitro* experiment results showed that CMSN@SRT@Anti could effectively reduce TC content in RAW264.7 cells. In addition, the TC lowering effect of CMSN@SRT@Anti was stronger than that of SRT1720 alone, indicating that CMSN@SRT@Anti with targeting ability can enhance the therapeutic effect of SRT1720 on AS [42]. Similar results were obtained in vivo. It is worth noting that there was no statistical difference in the anti-AS effect between CMSN@SRT and SRT1720 in vitro. However, the effect of SRT1720 was better than CMSN@SRT in vivo. This might be caused by the different environment between in vitro and in vivo. In *vitro*, the relatively static culture environment in the cell dish was conducive to the phagocytosis of macrophages, while in *vivo*, the nanomaterials were constantly flowing in the blood circulation, and the accumulation of CMSN@SRT without targeting ability were less in the lesions of AS, which was not conducive to the treatment of AS. In addition, due to the low dose of AntiCD36 contained in CMSN@SRT@Anti, the NMs failed to show the competitive inhibitory effect of AntiCD36 on the treatment of AS [21].

The metabolism of NMs in *vivo* is an important index to evaluate the safety of NMs. Studies have shown that the distribution and metabolism of NMs in organisms are related to the size of NMs and the characteristics of viscera. NMs with particle size less than 5.5 nm are usually metabolized through the urinary system, while NMs with particle size greater than 15 nm are not easy to pass through the glomerulus and are eventually excreted to the cholecyst through the liver [44]. The particle size of NMs synthesized in this study was larger than 15 nm. The results of fluorescence imaging in mice suggested that CMSN@Anti was excreted by liver and cholecyst. Fluorescence signals appeared in the liver half an hour post drug injection, and began to appear in the cholecyst at 1 h. Signals in the liver and cholecyst decreased to normal level at 24 h, indicating that the NMs had been basically expelled, which was in line with the characteristics of large-particle size NMs metabolism in vivo. During cell imaging, with or without ox-LDL stimulation, compared with CMSN@SRT, the uptake of CMSN@SRT@Anti by macrophages was significantly increased, indicating the targeted ability of AntiCD36 to RAW264.7 cells, which was consistent with the relevant description in the literature [19, 20]. In addition, compared with the untreated RAW264.7 cells, the uptake of CMSN@SRT@Anti in RAW264.7 cells after ox-LDL stimulation was significantly increased. This experimental phenomenon not only confirms the theory that ox-LDL can promote the upregulation of CD36 expression on macrophage surface [20], but also demonstrates that the NMs in this study have a better targeting effect on the lesion of acute advanced atherosclerosis. In vivo fluorescence imaging showed the targeting ability of CMSN@SRT@Anti to mouse aortic AS lesions via AntiCD36. Furthermore, the ApoE−/− mice were in good mental state during the 4 weeks of the NMs treatment, and no significant abnormalities were observed in organ pathology after treatment.

However, there are some limitations in this study. For example, the excitation visible fluorescent dye selected in this project has poor penetration, and its penetration in vivo is far less strong than that of near-infrared fluorescent dye, resulting in an increase in experimental consumables and labor costs for NMS imaging and metabolism detection in vivo.

## Conclusion

In the current study, we used mesoporous silicon as a nanoplatform to synthesize NMs for the precise diagnosis and treatment of atherosclerotic lesions. AntiCD36 was chosen as target molecule to recognize macrophages and SRT1720 loaded in mesoporous acted as the drug to inhibit the progress of AS plaque. It should be mentioned that such fabrication of functional NMs not only realized the tracking of AS lesions, but also enhanced the therapy efficacy of free SRT1720, which provided a new avenue for the applications of nanomaterials in the diagnosis and therapy of AS.

## Methods

### Synthesis and characterization of CMSN@SRT@Anti

The fabrication of the CMSN@SRT@Anti began with the preparation of Ru(bpy)_3_@SiO_2_. Typically, 3.5 mL of Ru(bpy)_3_Cl_2_ solution (10 mM in H_2_O) was added to a mixture of 35 mL cyclohexane, TritonX-100 and hexanol with a ratio of 4:1:1. The obtained mixture was maintained at room temperature for 30 min under magnetic stirring. Then, 1 mL mixed solution of TEOS and NH_3_^.^H_2_O with a ratio of 1:1 was added and was continued stirred for 24 h. At the end of the reaction, the mixed solution was centrifuged (6000 rpm, 30 min) to obtain a light red precipitate, named Ru(bpy)_3_@SiO_2_. The product was washed with ethanol for 3 times, and then dried in a lyophilizer and stored at 4 °C away from light.

The covering of mSiO_2_ layer was used for drug delivery in the current study. Briefly, 25 mL N-Hexadecyltrimethylammonium chloride (CTAC) aqueous solution (25% wt) and 250 μL triethylamine (TEA) were added into 35 mL Ru(bpy)_3_@SiO_2_ solution (3 mg/mL). After mixing evenly, 20 mL tetraethyl orthosilicate (TEOS) (5%, v/v) was added. The mixture was stirred at 60 °C (200 rpm) for 24 h without light. After the reaction, the obtained product was demoulded by NaCl-methanol solution (1% wt) (3 times, 24 h each time). The obtained CMSN was washed, dried and finally preserved at 4 °C in isolation from sunlight.

The CMSN@SRT@Anti was formed in three steps. Firstly, the amination of CMSN was performed. 20 μL (3-Aminopropyl) triethoxysilane (APTES) was dropped in 3 mL CMSN solution (10 mg/mL in anhydrous toluene) slowly, and they were stirred at 60 °C for 5 h under nitrogen protection. The precipitate was washed by anhydrous ethanol for three times and dried for later use. Secondly, SRT1720 was loaded. Briefly, 30 μL SRT1720 solution (50 mM in Dimethyl sulfoxide) were incubated with 470 μL amino modified CMSN (1.0 mg/mL) overnight in a thermostatic oscillator (37 °C, 100 rpm). After the reaction, the centrifugal precipitate was washed with PBS for three times to remove the unloaded drugs. Finally, covalent binding of CMSN@SRT with AntiCD36 was performed. Briefly, 4 μg AntiCD36 was dissolved in 1-(3-Dimethylaminopropyl)-3-ethylcarbodiimide hydrochloride (EDC) solution (2 mg/mL in 500 μL PBS) for 15 min for carboxyl group activation. Then, it was mixed with 500 μL solution containing 1 mg CMSN@SRT and 1 mg N-hydroxysuccinimide (NHS) for 2 h incubation at 37 °C. After centrifugation and washing, the precipitates were dispersed in 1 mL PBS and stored at 4 °C before use. The CMSN@Anti were prepared following the same procedures as described above, except without SRT1720 loading.

Ru(bpy)_3_@SiO_2_ and CMSN were characterized by TEM (TECNAI- G2, FEI, USA). The UV–vis absorption spectra and the fluorescence spectra were recorded by an ultra-micro spectrophotometer (NanoDrop One, Thermo Fisher Scientific, Waltham mass, USA) and a fluorescence spectrophotometer (LS-55, PerkinElmer, USA), respectively. FT-IR spectra were detected by Fourier transform infrared spectrometer (GB/T 2186-2007, Piketech, UK). The surface potential and hydrated particle size were detected by Zeta potentiometer (Nano ZS90, Malven, UK).

### Drug loading and drug release in vitro

In order to evaluate the drug loading and drug release of CMSN@SRT@Anti in vitro, the standard curve of SRT1720 was firstly determined with the UV–vis absorbance intensity of SRT1720 at 246 nm as the ordinate and the corresponding concentrations (0.01–5 mM) as the abscissa. Then, 0.5 mg CMSN-NH_2_ nanoparticles were mixed with different concentrations of SRT1720 (0.5, 1, 3, 5 mM, 500 μL) and the mixture was shaken overnight by an oscillator at 37 °C. The obtained solution was ultracentrifuged using a 100 kDa ultrafiltration cube and the unloaded SRT1720 amount in the filtrate was determined by UV–vis absorbance at 246 nm. Then, the AntiCD36 was conjugated with CMSN@SRT and the lost SRT1720 in this process was also determined to calculate the final SRT1720 loading amount in CMSN@SRT@Anti. The encapsulation efficiency (EE) and loading efficiency (LE) of CMSN@Anti for SRT1720 were calculated using the following equations:

$$ {\text{EE }}\left( \% \right) = \,\frac{{m_{SRT1720 , total} - m_{SRT1720, free} }}{{m_{SRT1720 , total} }} \times \,{1}00\%$$

$$ {\text{LE }}\left( \% \right) = \frac{{m_{{{\text{SRT}}1720{ },{\text{ total}}}} - m_{{{\text{SRT}}1720,{ }f{\text{ree}}}} }}{{m_{{{\text{SRT}}1720{\text{ loaded CMSN}}@{\text{Anti}}}} }}\, \times \,{1}00\%$$

The method of in *vitro* release is as follows. 0.5 mL CMSN@SRT@Anti (0.25 mg/mL) was transferred into a dialysis bag (MWCO = 100 KDa) and placed in tubes containing PBS with pH of 6.5, 7.4, or 9 respectively. They were rotated at 37 °C with a speed of 200 rpm for 48 h. 2 μL liquid in the tubes was picked up at scheduled time points, and their UV–vis absorbance values were measured using an ultra-micro spectrophotometer. The PBS buffer volume in the test tube remains constant.

### Cell culture

RAW264.7 (mouse macrophage cell line) and NIH-3T3 (mouse embryonic fibroblast cell line) were purchased from the Cell Bank of the Chinese Academy of Sciences (Shanghai, China). They were incubated with DMEM (Gibco, Logan, Utah, USA) supplemented with 10% fetal bovine serum (Thermo Fisher Scientific, Waltham mass, USA) and 1% Penicillin–Streptomycin (Biyuntian, Shanghai, China). The cells were maintained in a 5% CO_2_ incubator at 37 °C. For in *vitro* AS simulation, RAW264.7 cell were pretreated with 60 μg/mL ox-LDL (Yuanye Biotechnology, Shanghai, China).

### Biocompatibility evaluation in vitro and in vivo

The cytotoxic effect of CMSN@SRT@Anti on RAW264.7 or NIH-3T3 was evaluated by the MTT method. After the incubation of RAW264.7 or NIH-3T3 with different concentrations of nanomaterials in 96-well plates (n = 6) for 24 h (0–40 μg/mL), the media was removed. After washing with PBS, 100 μL 1 mg/mL MTT (Haoran Biotechnology, Shanghai, China) was added to each well. After further culture for 4 h, the MTT solution was discarded gently and 100 μL of Dimethyl sulfoxide (DMSO) (Pierce, Rockford, USA) was added to dissolve the formazan crystals. Finally, the absorbance of each well at 490 nm was measured with a microplate analyzer (Multiskon MK3, Thermo Fisher Scientific, Waltham mass, USA).

For in vivo biocompatibility evaluation, hematological and biochemical analyses of mouse with intraperitoneal injection of CMSN@SRT@Anti were carried out. The Kunming mice were treated with CMSN@SRT@Anti or an equivalent volume of PBS at a dose of 170 mg/kg/d every other day. Eyeball blood samples were taken at 1 day, 7 day and 21 day after nanomaterials injection as well as 21 day after PBS injection. The collected blood was used for blood biochemical detection (n = 3) and routine blood detection (n = 3). Hematoxylin–eosin (H&E) staining was performed for the main organs of the mice after different treatment and observed under an optical microscope.

To further study the metabolism of CMSN@SRT@Anti, the Kunming mice were injected intraperitoneally with the nanomaterials (170 mg/kg). The mice were sacrificed at scheduled time points (0, 0.5, 1, 2, 4, 6, 12, 24 h). Fluorescence accumulation in liver and cholecyst were semi-quantified by fluorescence imaging software (5200Multi, Ton, Shanghai, China).

### In vitro fluorescence imaging

To show the specific targeting ability of our fabricated CMSN@SRT@Anti to macrophages, RAW264.7, NIH-3T3, RAW264.7 pre-treated with 60 μg/mL ox-LDL or NIH-3T3 pre-treated with 60 μg/mL ox-LDL were first inoculated in 6-well plates, respectively (n = 3). Then, the cells were then incubated with CMSN@SRT@Anti or CMSN@SRT with a concentration of 16 μg/mL for 2 h at 37 °C. After fixed with 4% paraformaldehyde at room temperature for 15 min, the cells were washed twice with PBS and the nuclei were stained with DAPI. The cellular fluorescence imaging was observed under an inverted fluorescence microscope (DMI3000B, Leica, Germany).

### Western blot analysis

RAW264.7 cells were evenly inoculated into 6-well plates and divided into control group and ox-LDL-treated group (60 μg/mL) (n = 3). After different treatment, cells were harvested and total protein from cells was extracted by using Radio Immunoprecipitation Assay (RIPA) lysis buffer. After separated with sodium dodecyl sulfate-poly- acrylamide gel electrophoresis (SDS-PAGE) gels, samples were then transferred to nitrocellulose (NC) membrane. The membrane was blocked with 5% nonfat dried milk diluted in Tris buffered saline Tween (TBST) for 1 h at room temperature and then incubated with primary antibodies against β-actin and CD36 overnight at 4 ℃. After washed with TBST, the membranes were further incubated with corresponding secondary antibodies for 1 h at room temperature. Finally, the protein bands were detected on Tanon 5200 chemiluminescence imaging analysis system (Tanon, Shanghai, China) using efficient chemiluminescence (ECL) reagent and analyzed with the ImageJ software.

### The evaluation of inhibition effect of macrophage foaming in vitro

To further evaluate the therapy effect of CMSN@SRT@Anti in vitro, macrophage cells were treated with different groups: blank control group (BLK, RAW264.7 without any intervention), Model group (MG, RAW264.7 was incubated with ox-LDL only), CMSN group (RAW264.7 was incubated with ox-LDL and CMSN), CMSN@Anti group (RAW264.7 was incubated with ox-LDL and CMSN@Anti), CMSN@SRT group (RAW264.7 was incubated with ox-LDL and CMSN@SRT), CMSN@SRT@Anti group (RAW264.7 was incubated with ox-LDL and CMSN@SRT@Anti), free AntiCD36 group (RAW264.7 was incubated with ox-LDL and AntiCD36), and free SRT1720 group (RAW264.7 was incubated with ox-LDL and SRT1720). The concentrations of ox-LDL in all groups were 60 μg/mL. After different treatments, the TC contents in cells were quantitatively analyzed using the TC assay kit (Suoqiao Biology, Shanghai, China) (n = 3).

In order to more intuitively demonstrate the inhibitory effect of different treatments on the RAW264.7 cell foaming, oil red staining was carried out to instead TC value determination. ImageJ software was used to semi-quantitatively analyze the red stained area (n = 3).

The cell oil red O staining method was referred to the instruction. In brief, the treated cells were fixed with 4% paraformaldehyde (VICMED, Xuzhou, China) at room temperature for 15 min. After rinsed with PBS twice, the oil red working solution was added to stain for 40 min in the dark. Then, the cells were rinsed with distilled water for three times and observed under a fluorescent inverted microscope. The configuration of the oil red O working liquid was prepared as follows. 6 mL 0.5% saturated oil red O was mixed with 4 mL distilled water, and purified by filtering through a filter paper in the dark.

### Development of animal model

The purchased six week-old male ApoE-/-mice (male, GemPharmatech, Nanjing, China), initially fed a normal diet feeding 1 week to adapt to the new environment. Then, mice continued to be fed with the normal diet as a negative control group (low-fat diet, LFD) and a (high-fat diet, HFD) for 12 weeks to establish AS model. Composition of HFD is 40% Fat and 1.25% Cholesterol. Kunming male mice (Xuzhou Medical University, Xuzhou, China) were used to study the potential toxicity and metabolism of our prepared nanomaterials. All animal procedures followed the regulations of the Chinese Physiological Society for the Management of Laboratory Animals. The experimental scheme was in line with the ethical requirements of the Animal Care Committee of Xuzhou Medical University (202008A077).

### In vivo targeted fluorescence imaging

In order to select the best imaging time point of nanoparticles in vivo, 5 male AS-model mice (HFD for 16 w) with similar weight and status were used. 4 of them were treated with intraperitoneal injection with CMSN@SRT@Anti (170 mg/kg) respectively. The mice were sacrificed at different time points (0, 1, 4, 8, 24 h), and the isolated aortas were collected. Then, the arteries were washed with saline three times and visualized using a small animal fluorescence imaging system.

To further compare the specific targeting ability of CMSN@SRT@Anti and CMSN@SRT, the AS-model mice were intraperitoneal injection with PBS, CMSN@SRT@Anti or CMSN@SRT with an amount of 170 mg/kg (n = 3), respectively. Four hours later, the isolated aortas were washed with saline and placed in a small animal fluorescence imaging system. The signal intensities of whole arteries were quantified using Tanon Image Gel software (Tanon, Shanghai, China).

### In vivo* therapy*

To evaluate the therapy effect of AS by CMSN@SRT@Anti in vivo, ApoE−/−mice (HFD for 12 w) were divided into five groups(n = 4): the LFD + PBS group (LFD), the HFD + PBS group (Vehicle), the HFD + CMSN@SRT@Anti, the HFD + CMSN@SRT, and the HFD + free SRT1720 group (SRT1720). AS-model mice fed with HFD were intraperitoneal injected with nanomaterials (170 mg/kg/d) or SRT1720 (50 mg/kg/d) [42] or the same volume of PBS liquid every other day for 4 weeks. The mice were sacrificed after the treatment. The TC values in blood were determined by the TC determination kit, and the aorta red staining areas after oil red staining were analyzed semi-quantitatively by ImageJ. H&E was introduced to show the changes of the main organs of mice after CMSN@SRT@Anti treatment.

The Oil Red O staining procedure to observe the plaque of lipid deposits was as follows. The aorta was dissected, and the adventitial fat was removed after being opened longitudinally with the luminal surface toward the outside. After fixed in 4% paraformaldehyde at room temperature for 2 h, the aorta was placed in 0.3% Oil Red O working solution (Solarbio, Beijng, China) at 45 ℃ for 1 h in the dark condition, and then rinsed with 85% isopropanol for 3 times. The ImageJ quantitatively analyzed the aorta (thoracic aorta, abdominal aorta and aortic arch).

### Statistical analysis

All the analyses involved in this project were performed by SPSS 22.0 software. Data were expressed as mean ± standard deviation. Shapiro–Wilk test was used to determine the normality of the data distribution. The multi-group comparisons were made with a one-way ANOVA analysis, followed by Dunnett’s post hoc test. The Student’s *t* test was used for comparison between the two groups. According to the test level α = 0.05, *P* < 0.05 was considered to be statistically significant.

## Supplementary Information

**Additional file 1:** **Fig. S1 a.** The fluorescence intensity values of Ru(bpy)_3_Cl_2_ and CMSN at various time points under the UV lamp. **b** The hydrated particle size of CMSN@Anti. (The mean particle sizes of NPs were 225.3 nm, 231.6 nm, 267.8 nm and 255.4 nm respectively). **Fig. S2 a.** The UV-vis absorption spectra of SRT1720 at different concentrations. **b** The corresponding linear regression equation. **Fig. S3.** Toxicity test of CMSN@SRT@Anti to RAW264.7 or NIH-3T3 cells (*F*_RAW264.7_ = 1.679, *P *= 0.142 > 0.05; *F*_NIH-3T3_ = 1.737, *P* = 0.128 > 0.05). Fig. S4. Representative blots and quantification of protein levels of CD36 to the β-actin levels before and after ox-LDL treatment. (*p < 0.05). **Fig. S5.** Blood index analysis of Kunming mice before and after CMSN@SRT@Anti intervention. ALT (P=0.624), TP (P=0.972), ALB (*P*=0.504), CREA (*P*=0.577), UREA (*P*=0.849), WBC (*P*=0.984), RBC (*P*=0.664), HGB (*P*=0.775), HCT (*P*=0.707), MCH (*P*=0.965), MCV (P=0.963), *P* values are all greater than 0.05. Compared with AST _0d_ group, P(AST _1d_)= 0.023<0.05, P (AST _7d_)= 0.448>0.05, P (AST _21d_)= 0.313>0.05. **Fig. S6.** Toxicity test of CMSN@SRT@Anti on Kunming mice. a The body weight of Kunming mice injected intraperitoneally with NMs or PBS changed with time. b Pathological section of major organs in Kunming mice (H&E, Scale bar: 100 μm). **Table S1.** EE and LE of CMSN@SRT@Anti at different concentrations of SRT1720. **Table S2.** Relative fluorescence intensity of liver and cholecyst of Kunming mice at each time point after intraperitoneal injection of CMSN@SRT@Anti determined by Tanon Image softerware.

## Data Availability

All data generated or analysed during this study are included in this published article and its Additional file 1.

## Abbreviations

AS: Atherosclerosis
CHD: Coronary heart disease;
CVD: Cardiovascular disease
HFD: High-Fat diet
LDL: Low-density lipoprotein
MSN: Mesoporous silica nanoparticle
NMs: Nanomedicines
Ox-LDL: Oxidized low-density lipoprotein
TC: Total cholesterol
EE: Encapsulation efficiency
LE: Loading efficiency
TEOS: Tetraethoxysilane
APTES: Triethoxysilane
DMSO: Dimethyl sulfoxide
VSMCs: Vascular smooth muscle cells
PFN1: Profilin-1 Antibody
SV40: Simian virus 40
MMPs: Matrix metalloproteinases
TEM: Transmission Electron Microscopy
UV–vis: Ultraviolet–visible
FT-IR: Fourier transform infrared spectroscopy
VASPs: Vulnerable atherosclerotic plaques
SDS-PAGE: Sodium dodecyl sulfate-poly- acrylamide gel electrophoresis
TBST: Tris buffered saline Tween
ECL: Efficient chemiluminescence
RIPA: Radio Immunoprecipitation Assay
