# Supplementary material for: Theranostic nanoplatform to target macrophages enables the inhibition of atherosclerosis progression and fluorescence imaging of plaque in ApoE(−/−) mice
Source: J Nanobiotechnology. 2021 Jul 28;19:222. doi: 10.1186/s12951-021-00962-w (PMC8317354; doi:10.1186/s12951-021-00962-w)
Supplement: Supplementary file 1 — Additional file 1: Fig. S1 a. The fluorescence intensity values of Ru(bpy)3Cl2 and CMSN at various time points under the UV lamp. b The hydrated particle size of CMSN@Anti. (The mean particle sizes of NPs were 225.3 nm, 231.6 nm, 267.8 nm and 255.4 nm respectively). Fig. S2 a. The UV-vis absorption spectra of SRT1720 at different concentrations. b The corresponding linear regression equation. Fig. S3. Toxicity test of CMSN@SRT@Anti to RAW264.7 or NIH-3T3 cells (FRAW264.7 = 1.679, P = 0.142 > 0.05; FNIH-3T3 = 1.737, P = 0.128 > 0.05). Fig. S4. Representative blots and quantification of protein levels of CD36 to the β-actin levels before and after ox-LDL treatment. (*p < 0.05). Fig. S5. Blood index analysis of Kunming mice before and after CMSN@SRT@Anti intervention. ALT (P=0.624), TP (P=0.972), ALB (P=0.504), CREA (P=0.577), UREA (P=0.849), WBC (P=0.984), RBC (P=0.664), HGB (P=0.775), HCT (P=0.707), MCH (P=0.965), MCV (P=0.963), P values are all greater than 0.05. Compared with AST 0d group, P(AST 1d)= 0.023<0.05, P (AST 7d)= 0.448>0.05, P (AST 21d)= 0.313>0.05. Fig. S6. Toxicity test of CMSN@SRT@Anti on Kunming mice. a The body weight of Kunming mice injected intraperitoneally with NMs or PBS changed with time. b Pathological section of major organs in Kunming mice (H&E, Scale bar: 100 μm). Table S1. EE and LE of CMSN@SRT@Anti at different concentrations of SRT1720. Table S2. Relative fluorescence intensity of liver and cholecyst of Kunming mice at each time point after intraperitoneal injection of CMSN@SRT@Anti determined by Tanon Image softerware. [file 12951_2021_962_MOESM1_ESM.docx]

**Additional file 1 (Supplementary Figure)**

Qi Wang^1†^, Yong Wang^1†^, Siwen Liu^1^, Xuan Sha^1^, Xiaoxi Song^1^, Yue Dai^1^, Mingming Zhao^1^, Lulu Cai^1^, Kai Xu^1,2*^, Jingjing Li^1,2*^ID

^*^Correspondence: [qingchao0124@163.com](mailto:qingchao0124@163.com) ; [xkpaper@163.com](mailto:xkpaper@163.com)

^†^Qi Wang and Yong Wang contributed equally to this work

^1^ School of Medical Imaging, Xuzhou Medical University, Xuzhou 221006, China
^2^Department of Radiology, Affiliated Hospital of Xuzhou Medical University, Xuzhou 221004, China

**
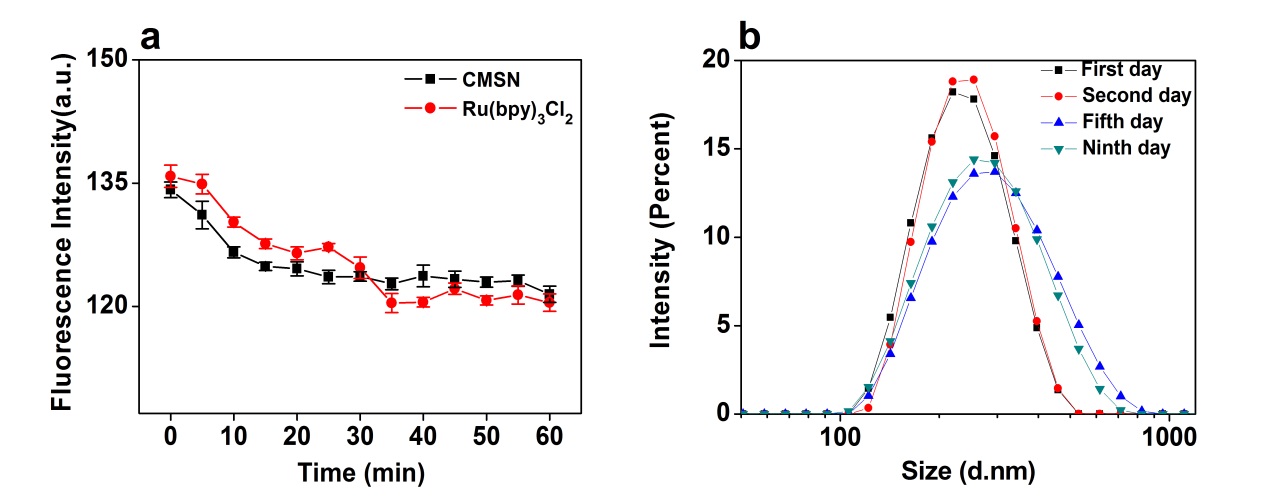
**

**Fig. S1 a** The fluorescence intensity values of Ru(bpy)_3_Cl_2_ and CMSN at various time points under the UV lamp. **b** The hydrated particle size of CMSN@Anti. (The mean particle sizes of NPs were 225.3 nm, 231.6 nm, 267.8 nm and 255.4 nm respectively).


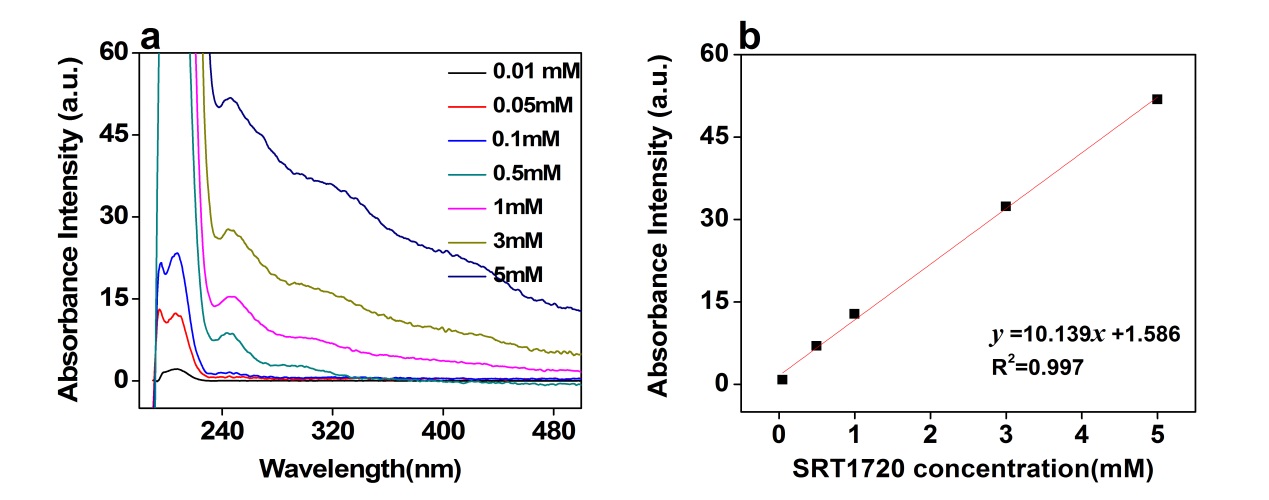


**Fig. S2 a** The UV-vis absorption spectra of SRT1720 at different concentrations. **b** The corresponding linear regression equation.


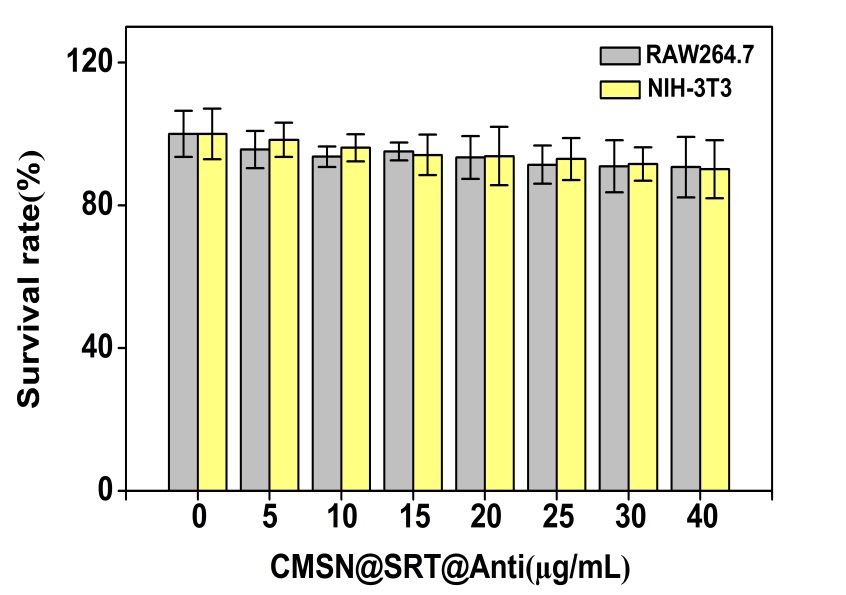


**Fig. S3** Toxicity test of CMSN@SRT@Anti to RAW264.7 or NIH-3T3 cells (*F*_RAW264.7_ = 1.679, *P* = 0.142 ＞ 0.05; *F*_NIH-3T3_ = 1.737, *P* = 0.128 ＞ 0.05).


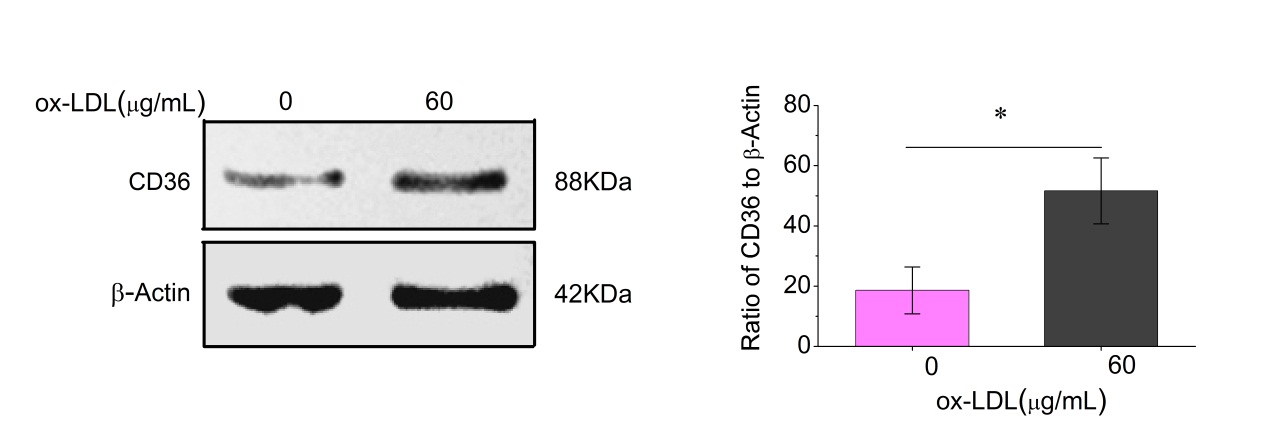


**Fig. S4** Representative blots and quantification of protein levels of CD36 to the β-actin levels before and after ox-LDL treatment.（**p*< 0.05）


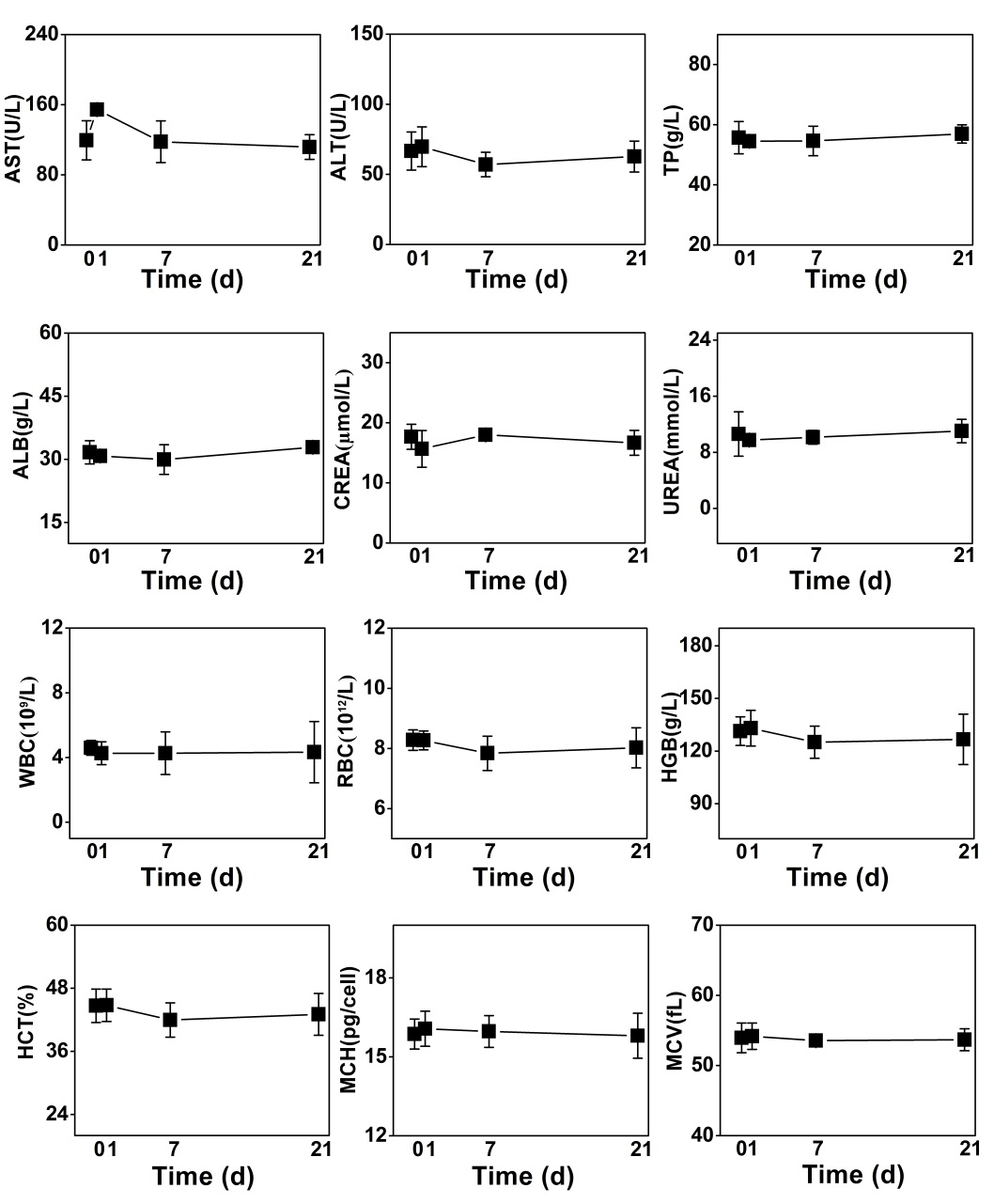


**Fig. S5** Blood index analysis of Kunming mice before and after CMSN@SRT@Anti intervention. ALT (P=0.624), TP (P=0.972), ALB (*P*=0.504), CREA (*P*=0.577), UREA (*P*=0.849), WBC (*P*=0.984), RBC (*P*=0.664), HGB (*P*=0.775), HCT (*P*=0.707), MCH (*P*=0.965), MCV (P=0.963), *P* values are all greater than 0.05.

Compared with AST _0d_ group, P(AST _1d_)= 0.023＜0.05, P (AST _7d_)= 0.448>0.05, P (AST _21d_)= 0.313>0.05.


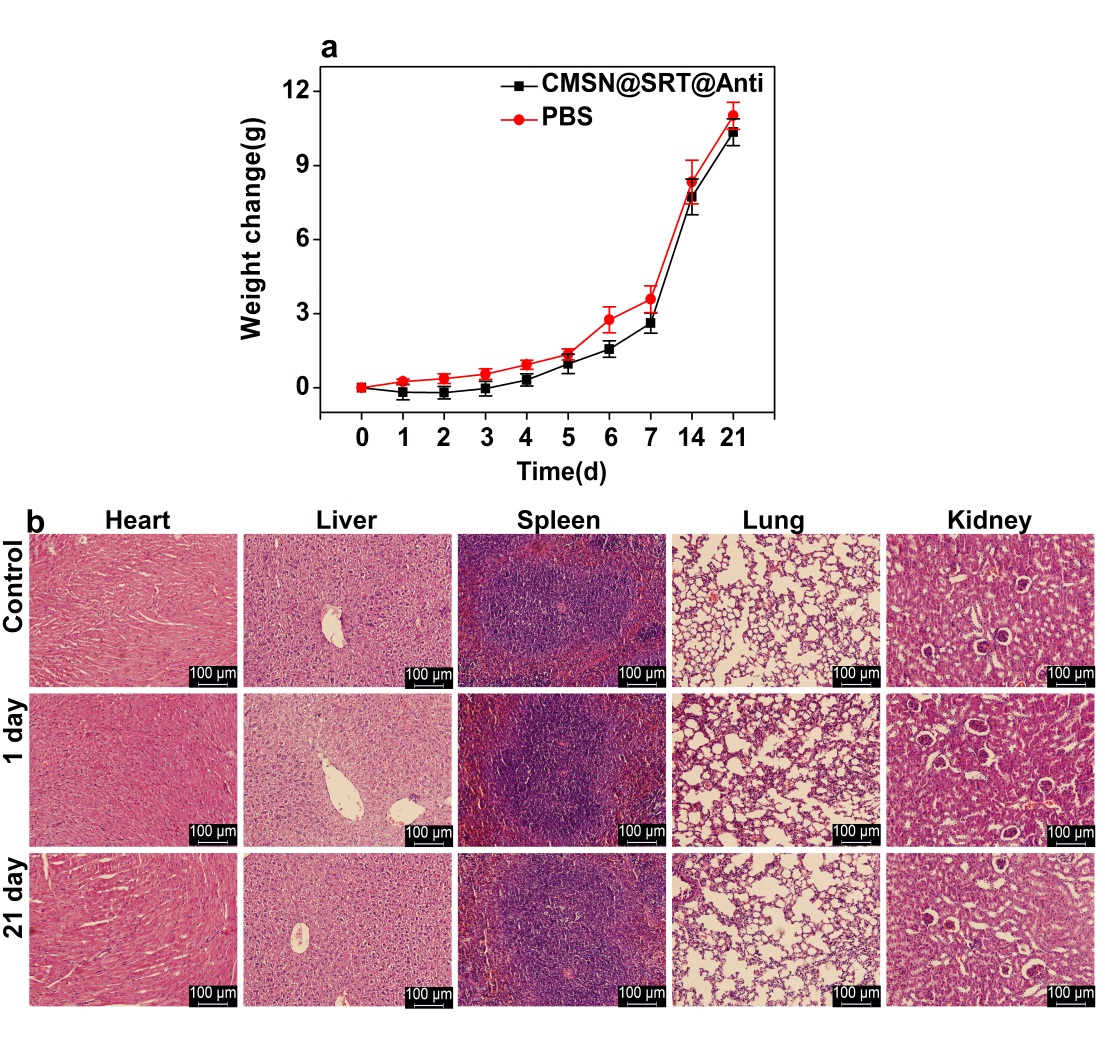


**Fig. S6** Toxicity test of CMSN@SRT@Anti on Kunming mice. **a** The body weight of Kunming mice injected intraperitoneally with NMs or PBS changed with time. **b** Pathological section of major organs in Kunming mice (H&E, Scale bar: 100 μm).

**Tab. S1** EE and LE of CMSN@SRT@Anti at different concentrations of SRT1720

| SRT1720 (mM) | Loading Efficiences (%) | Encapsulation Efficiences (%) |
| --- | --- | --- |
| 0.5 | 185) | 83±8 |
| 1 | 24±1 | 63±3 |
| 3 | 42±2 | 47±4 |
| 5 | 53±1 | 44±2 |

**Tab. S2** Relative fluorescence intensity of liver and cholecyst of Kunming mice at each time point after intraperitoneal injection of CMSN@SRT@Anti determined by Tanon Image softerware.

| Tissue | 0 h | 0.5 h | 1 h | 2 h | 4 h | 6 h | 12 h | 24 h |
| --- | --- | --- | --- | --- | --- | --- | --- | --- |
| Liver | 32.8 | 89.4 | 128.5 | 139.6 | 128.5 | 105.1 | 103.4 | 30.5 |
| Cholecyst | 27.9 | 34.7 | 159.5 | 176.8 | 240 | 195.1 | 143.6 | 33.4 |
